# Immediate Pre-Partum SARS-CoV-2 Status and Immune Profiling of Breastmilk: A Case-Control Study

**DOI:** 10.3389/fimmu.2021.720716

**Published:** 2021-07-26

**Authors:** Laura Sánchez García, Natalia Gómez-Torres, Fernando Cabañas, Raquel González-Sánchez, Manuela López-Azorín, M. Teresa Moral-Pumarega, Diana Escuder-Vieco, Esther Cabañes-Alonso, Irma Castro, Claudio Alba, Juan Miguel Rodríguez Gómez, Adelina Pellicer

**Affiliations:** ^1^ Neonatology Department, Biomedical Research Foundation-IDIPAZ, La Paz University Hospital, Madrid, Spain; ^2^ Nutrition and Food Science Department, Complutense University, Madrid, Spain; ^3^ Neonatology Department, Quironsalud Madrid University Hospital and Quironsalud San José Hospital, Biomedical Research Foundation-IDIPAZ, La Paz University Hospital, Madrid, Spain; ^4^ Neonatology Department, Quironsalud Madrid University Hospital and Quironsalud San José Hospital, Madrid, Spain; ^5^ Neonatology Department, 12 Octubre University Hospital, Madrid, Spain; ^6^ Neonatology Department and Regional Human Milk Bank, 12 Octubre University Hospital, Madrid, Spain

**Keywords:** COVID-19, vertical infectious disease transmission, breastfeeding, immunologic factors, immune system

## Abstract

**Objetive:**

To address the prevalence of SARS-CoV-2 and the evolutionary profile of immune compounds in breastmilk of positive mothers according to time and disease state.

**Methods:**

Forty-five women with term pregnancies with confirmed non-severe SARS-CoV-2 infection (case group), and 96 SARS-CoV-2 negative women in identical conditions (control group) were approached, using consecutive sample. Weekly (1st to 5th week postpartum) reverse transcription polymerase chain reaction (RT-PCR) in nasopharyngeal swabs (cases) and breastmilk (cases and controls) were obtained. Concentration of cytokines, chemokines, and growth factors in breastmilk (cases and controls) were determined at 1st and 5th week post-partum.

**Results:**

Thirty-seven (study group) and 45 (control group) women were enrolled. Symptomatic infection occurred in 56.8% of women in the study group (48% fever, 48% anosmia, 43% cough). SARS-CoV-2 RNA was not found in breastmilk samples. Concentrations of cytokines (IFN-γ, IL-1ra, IL-4, IL-6, IL-9, IL-13, and TNF-α) chemokines (eotaxin, IP-10, MIP-1α, and RANTES) and growth factors (FGF, GM-CSF, IL7, and PDGF-BB) were higher in breastmilk of the study compared with the control group at 1st week postpartum. Immune compounds concentrations decreased on time, particularly in the control group milk samples. Time of nasopharyngeal swab to become negative influenced the immune compound concentration pattern. Severity of disease (symptomatic or asymptomatic infection) did not affect the immunological profile in breast milk.

**Conclusions:**

This study confirms no viral RNA and a distinct immunological profile in breastmilk according to mother’s SARS-CoV-2 status. Additional studies should address whether these findings indicate efficient reaction against SARS-CoV-2 infection, which might be suitable to protect the recipient child.

## Introduction

During the first months of COVID-19 pandemic, some concerns arose about the safety of breastfeeding because of the potential risk of viral transmission. However, most of the human milk samples assayed for SARS-CoV-2 RNA Reverse Transcription Polymerase Chain Reaction (RT-PCR) have yielded negative results (1–5), whereas no evidence of SARS-CoV-2 transmission through human milk has been provided yet (6, 7).

With regard to the efficacy of breastmilk to provide protecting anti-SARS-CoV-2 antibodies (3, 8, 9), most studies carried so far have addressed their presence. However, information regarding the impact of COVID-19 on other immune compounds, such as cytokines, chemokines, and growth factors, is lacking. These immune factors act in the prevention of infantile infection and can modulate the immunological development of the infant (10–15). In fact, their abundance in human milk is often inversely related to their scarcity in the infant’s gut, characterized by a deficit of mucosal-related anti-inflammatory mechanisms, a limited production of secretory IgA, and a poor innate effector cell function (15).

Activation of inflammatory signaling pathway is a critical mediator in the pathophysiology of COVID-19 (16–18), and maternal environmental factors, including viral infections and previous antigenic exposures, are known to affect immunological composition of human milk (19–23). Therefore, a deeper insight on the impact of SARS-CoV-2 infection on the composition of breastmilk is needed.

This research aims to address questions related on the safety and the efficacy of breastmilk feeding of neonates born to mothers with non-severe SARS-CoV-2 infection, through the systematic assessment of: (a) the prevalence of viral RNA in breastmilk according to SARS-CoV-2 status, (b) the impact of SARS-CoV-2 infection on the milk profile of cytokines, chemokines, and growth factors, and (c) the evolution of their concentrations during the first five weeks of lactation.

## Methods

This multicenter, prospective case-control study was conducted in Madrid, between April and July 2020. Since March 2020, maternity hospitals tested pregnant woman for SARS-CoV-2 infection by routine nasopharyngeal RT-PCR as screening prior delivery. Given the high incidence of COVID-19 disease at the start of the study, the Spanish Ministry of Health considered a patient had confirmed SARS-CoV-2 infection whenever the RT-PCR was positive, regardless of clinical features. Four level 3 institutions of the health system of the Madrid region (Spain) (La Paz University Hospital, 12 de Octubre University Hospital, Quironsalud Madrid University Hospital and Quironsalud San José Hospital) participated in this study. The protocol was approved by the reference Clinical Research Ethics Committee. Informed consent was obtained from mothers before enrolment. Every mother-infant’s information was treated anonymously.

### Eligibility Criteria

Women with term pregnancies with confirmed SARS-CoV-2 infection at the time of delivery, who were in good clinical condition and had a decision to breastfeed were considered eligible for the study (study group). For each positive case, two consecutive women with term pregnancies, in identical conditions, who were SARS-CoV-2 negative were approached (control group). Prospective data recording of participant mothers (age, underlying pathology, type of delivery, time of positive SARS-CoV-2 RT-PCR and related clinical features/treatment) and their infants (gestational age, birth weight, neonatal diagnoses) were obtained.

### Study Procedures

During the first month after delivery, breastmilk (case and control groups) and nasopharyngeal swabs (case group) were collected by the participant mothers, who were instructed on accomplishment and storage of samples. Breastmilk samples were collected every 72 h from delivery after careful hand, breast, and nipple hygiene, with the mouth and nose covered by a mask. Milk was collected either by pump or manual extraction, and kept in individual sterile container for each aliquot. After milk extraction, breast pump was cleaned with soap and water, and disinfected by alcohol or immersion in boiling water. Case group mothers self-performed weekly nasopharyngeal smear using swab kits and the corresponding RT-PCR transport medium. Control group mothers underwent a serological study prior to hospital discharge. Blood samples were centrifuged and stored for analysis. Presence of IgG and IgM antibodies against SARS-CoV-2 was assessed using the IgG+IgM Combo Detection Kit (SD Biosensor, Korea).

All biological samples were identified by a study code and date of extraction, immediately frozen at −20°C, periodically collected at home by a specialized transport system and shipped on dry ice (−78.5°C) to the Nutrition and Food Science Department, Complutense University of Madrid where the samples were analyzed. To eliminate or minimize potential lab biases, all the samples were submitted to a single freeze–thaw cycle and were analyzed by the same researchers. After hospital discharge, follow up of the infants was done by serial phone calls during the first month of age.

#### RT-PCR Assays

RNA extraction from the nasopharyngeal and milk samples (200 μl) was carried out using the KINGFISHER FLEX 96 extraction robot (ThermoFisher), the MagMax_Core_Flex extraction program and the MagMAX Viral/Pathogen II Nucleic Acid Isolation kit (Applied Biosistems, ThermoFisher). For the detection of SARS-CoV-2, the TaqPath COVID-19 CE-IVD RT-PCR kit (Applied Biosystems, Thermo Fisher Scientific) was used in a 384-well format with the QuantStudio 7 Flex System equipment (Applied Biosystems). All procedures were performed following the manufacturer’s instructions.

#### Immunoassays in Breastmilk Samples

Concentration and frequency of detection of 30 soluble immune factors in the milk samples were determined by magnetic bead-based multiplex immunoassays using a Bioplex 200 instrument (Bio-Rad, Hercules, CA, USA) and the pro-human cytokine 27-plex assay (Bio-Rad). The immune factors included in this study were interleukin (IL) 1β, IL1ra, IL2, IL4, IL5, IL6, IL7, IL8, IL9, IL10, IL12(p70), IL13, IL15, and IL17, interferon-gamma (IFN-γ), granulocyte colony stimulating factor (G-CSF), granulocyte-macrophage colony stimulating factor (GM-CSF), monocyte chemoattractant protein-1 (MCP-1), macrophage inflammatory protein 1α and 1β (MIP-1α, MIP-1β), eotaxin, basic fibroblast growth factor (Basic FGF), tumor necrosis factor-alpha (TNF-α), inferon γ-induced protein (IP-10), platelet-derived growth factor-BB (PDGF-BB), regulated on activation normal T-cells expressed and secreted (RANTES), and vascular endothelial growth factor (VEGF). In addition, levels of transforming growth factor-beta 2 (TGF-β2), epidermal growth factor (EGF), and growth-related oncogene-α (Groα) were measured, respectively, by the human TGF-β2, human EGF, and human GRO alpha (CXCL1) ELISA kits (RayBiotech, Norcross, GA, USA).

To avoid interferences, the fatty layer and the somatic cells were removed from the milk samples. Briefly, sample aliquots (1 ml) were centrifuged at 11,000g for 15 min at 4°C, the intermediate aqueous phase was collected and stored at −20°C until analysis. Every assay was run in duplicate according to the manufacturer’s instructions using the same reagents’ batches and equipment; standard curves were performed for each analyte in every assay. All the concentrations were expressed as nanograms per liter (ng/L), except IP-10, VEGF, TGFβ2, EGF, and Groα, which were expressed as micrograms per liter (μg/L).

### Statistical Analysis

Demographic data with normal distribution were presented as the mean and standard deviation (SD). Regarding immune factors, normality of data distribution was examined through visual inspection of histograms and Shapiro-Wilks tests, both evidencing a non-normal distribution for all tested parameters (*ρ* < 0.05). Accordingly, nonparametric statistical analyses were performed, and data were expressed as the median and interquartile range (IQR). Immune factor concentrations were logarithmically transformed prior to statistical analysis. Differences in the relative abundance of the immune compounds were compared by Wilcoxon rank test and Mann-Whitney *U* test. To compare multiple comparisons, Bonferroni-adjusted *post hoc* significance levels were performed. Fisher’s exact probability test was performed to compare the frequency of detection of different immunological compounds. Significance was declared at *ρ* < 0.05 for all analyses. All analyses were performed with the R software version 4.0.3 (R-project, http://www.r-project.org). For the purpose of this report, immune factor concentration on one of the milk samples obtained during week 1 (day 3 to day 6) and week 5 (beyond day 28) postpartum were used for comparisons.

## Results

**Figure 1:**
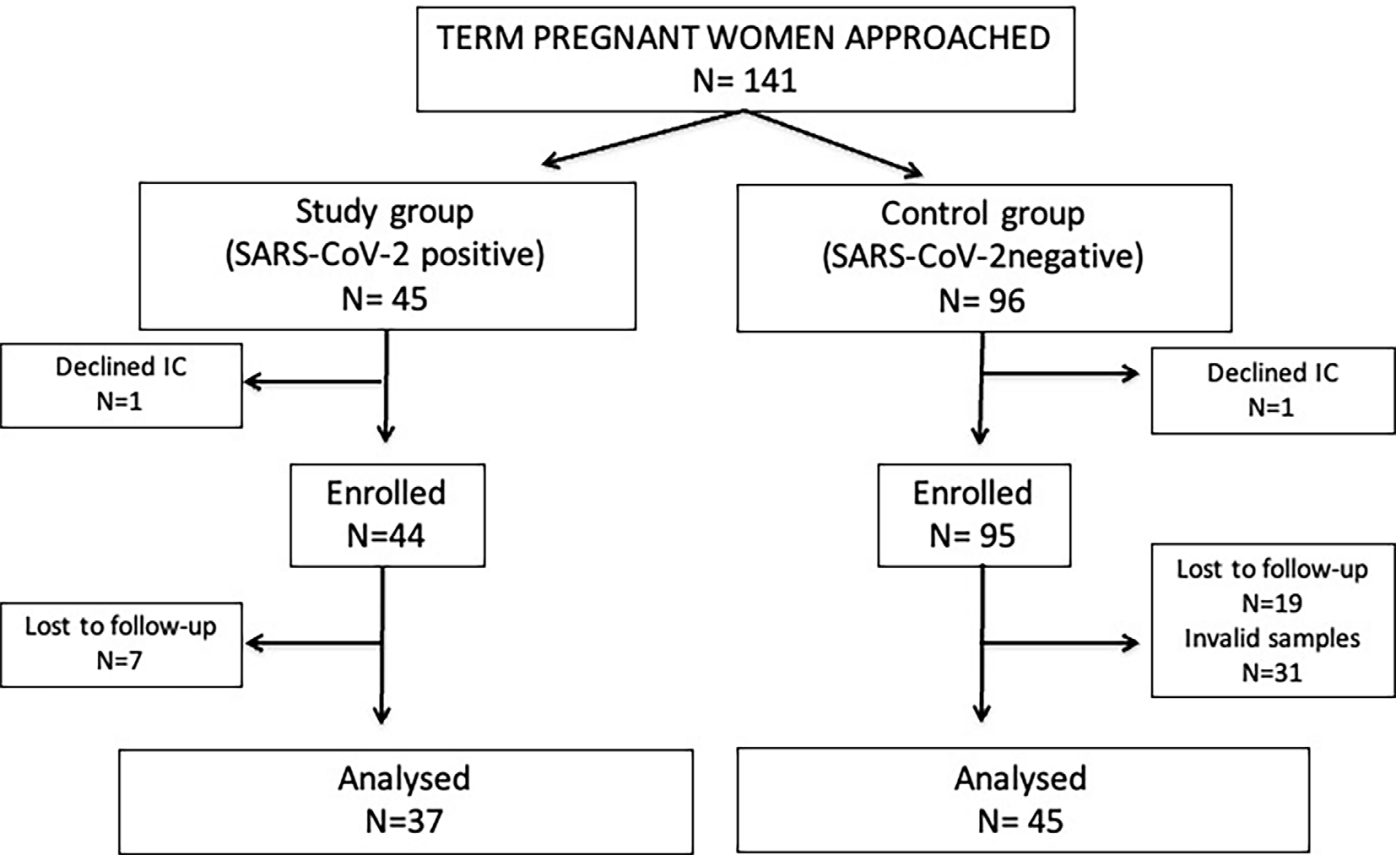
Flow diagram of participants. IC, informed consent; Invalid samples, non-availability of all samples to be compared, inadequate sample identification or conservation.

During the study period a total of 141 term-pregnant women who fulfilled eligibility criteria were approached. Of them, 37 study group and 45 control group women were included in the final analyses. Details on participants’ chart flow and reasons for exclusion are described in **Figure 1**.

No differences in maternal age [33.9 (5.4) *vs* 34.5 (5.1) years, p=0.612], previous maternal health problems prevalence (19% *vs* 13%, p= 0.493, only 1 case of obesity in study group), rates of vaginal delivery (73% *vs* 89%, p= 0.064), gestational age at birth [39.1 (1.8) *vs* 39.1 (1.6) weeks, p= 0.852], or birth weight [3187 (543) *vs* 3240 (469) grams, p= 0.639] between study and control group were found.

By hospital protocol, nasopharyngeal PCR was performed at 24 h and at 36 to 48 h from birth on infants of positive SARS-COV-2 mothers, resulting negative in all cases. None of infants of mothers in study and control group presented clinical signs of SARS-COV-2 infection in the first month of life.

Among the study group, 21 (56.8%) women presented mild SARS-CoV-2 infection related symptoms, consisting of fever (48%), anosmia (48%), cough (43%), ageusia (14%), odynophagia (10%), myalgia (10%), diarrhea (10%), or headache (5%). Nineteen (51.3%) received medication (anticoagulation, antibiotics, hydroxychloroquine, oxygen therapy) around labor. Serological analyses of control women were negative.

### RT-PCR Assays

Nasopharyngeal RT-PCR tests were serially conducted in 30 of the 37 SARS-CoV-2–positive women [four samples (1 per week), n=25; three samples (weeks 1–3), n=5; no samples, n=7]. Nasopharyngeal RT-PCR tests attained negative results at week 2 (n=7, 23.3%), at week 3 (n=9, 30%), and at week 4 (n=9, 30%) postpartum and remained positive at the last sample that was tested in 5 (16.6%) participants (3 at week 3, and 2 at week 4).

All human milk samples analyzed were negative for SARS-CoV-2 RNA as assessed by RT-PCR.

### Immunological Assays in Breastmilk Samples

All of the 30 immunological factors that were searched for in breastmilk could be detected in, at least, some of the milk samples. IFN-γ, IL-8, IL-12(p70), IL-17, IP-10, MIP-1β, TNF-α, VEGF, TGFβ2, EGF, and GROα displayed the highest frequencies of detection (100% of the samples), closely followed by eotaxin, G-CSF, IL-1β, IL-1ra, IL-2, IL-4, IL-6, IL-7, IL-9, and RANTES, which were detected in >95% of the samples. In contrast, IL-5 and IL-15 were the least frequently detected compounds (≤70% of the samples). No differences were observed between samples collected in week 1 or week 5 postpartum, with the exception of GM-CSF, which frequency of detection in week 1 (79%) was lower than that in week 5 (90%) (*p*=0.047).

#### Breastmilk Cytokine Pattern

**Table 1** displays concentration of cytokines in breastmilk samples. IFN-γ, IL-1ra, IL-4, IL-6, IL-9, IL-13, and TNF-α showed higher concentrations in study than in the control group at both sampling times. In addition, IL-1β and IL-2 at week 5 postpartum were higher in breastmilk samples of the study group.

**Table 1:** Concentration (ng/l) of cytokines in milk samples of study and control group women over time.

Week 1 postpartum				Week 5 postpartum				
	STUDY GROUP (n = 36)	CONTROL GROUP (n = 45)		STUDY GROUP (n = 37)	CONTROL GROUP (n = 45)			
Immune Factors	Median (IQR)	Median (IQR)	*ρ* ^a^	Median (IQR)	Median (IQR)	^b^ *ρ*	^c^ *ρ*	^d^ *ρ*
IFN- γ	160.5 (78.8-301.8)	50.4 (23.7-117.7)	0.003	135.8 (18.3-151.6)	11.8 (4.3-23)	<0.001	<0.001	<0.001
IL-1β	3.2 (1.5-5.0)	2.1 (1.4-3.7)	0.273	2.7 (1.8-3.3)	1.0 (0.7-1.4)	<0.001	0.117	<0.001
IL-1ra	5966.4 (1642.5-62227.1)	1444.3 (676.2-3939.5)	0.023	5896.3 (662.9-5979.4)	443.6 (271.9-852.4)	<0.001	0.004	<0.001
IL-2	5.8 (3.8-7.8)	4.2 (3.0-6.1)	0.164	4.7 (2.4-6.7)	3.1 (2.3-3.8)	0.014	0.300	0.002
IL-4	3.9 (1.3-3.7)	1.0 (0.6-2.1)	0.001	2.6 (1.6-2.7)	0.5 (0.3-0.7)	<0.001	0.007	<0.001
IL-6	208.5 (28.5-223.7)	17.7 (7.5-46.2)	<0.001	207.1 (21.1-212.1)	3.6 (2.3-10.9)	<0.001	0.075	<0.001
IL-9	29.8 (14.8-40.8)	13.1 (8.6-20.0)	0.005	27.3 (11.1-29.7)	5.4 (3.9-8.4)	<0.001	0.041	< 0.001
IL-10	3.3 (1.8-4.8)	3.7 (2.0-4.7)	0.795	2.9 (1.9-4.2)	3.3 (2.7-4.3)	0.363	0.279	0.565
IL-12 (p70)	3.9 (3.0-4.5)	3.8 (3.4-4.3)	0.890	3.4 (2.8-4.1)	3.8 (3.4-4.1)	0.052	0.505	0.712
IL-13	1.4 (0.9-1.9)	0.9 (0.7-1.3)	0.007	1.4 (0.8-1.9)	0.9 (0.7-1.3)	0.043	0.260	0.756
IL-15	94.3 (59.9-164.7)	99.2 (82.6-137.2)	0.921	90.7 (67.5-149.9)	90.7 (49.1-128.3)	0.560	0.734	0.039
IL-17	13.2 (5.4-15.9)	11.3 (10.4-16.7)	0.436	11.8 (5.0-15.3)	9.7 (8.1-12.5)	0.567	0.915	0.007
TNF-α	106.3 (67.9-130.2)	35.5 (25.9-75.8)	0.001	92.2 (73.0-112.4)	17.7 (11.5-50.8)	<0.001	0.314	<0.001

a ρ: Mann-Whitney U test was used to evaluate differences in concentration of cytokines between milk samples from STUDY GROUP and CONTROL GROUP collected in week 1 postpartum.

b ρ: Mann-Whitney U was used to evaluate differences in concentration of cytokines between milk samples STUDY GROUP and CONTROL GROUP collected in week 5 postpartum.

c ρ: Wilcoxon signed rank test was used to evaluate differences in the concentration of cytokines of the milk samples of STUDY GROUP between week 1 and week 5 postpartum.

d ρ: Wilcoxon signed rank test was used to evaluate differences in the concentration of cytokines of the milk samples of CONTROL GROUP between week 1 and week 5 postpartum.

**Figure 2:**
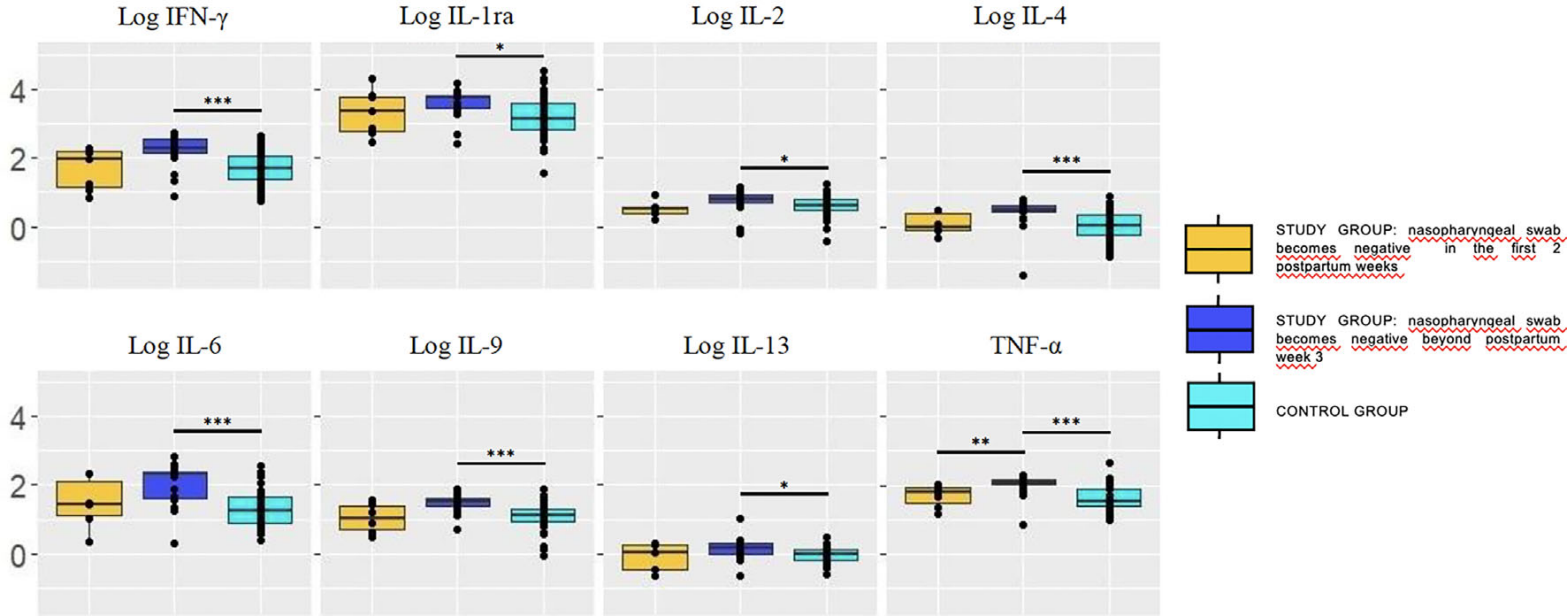
Concentration (log) of cytokines in breastmilk of study group women according to the moment in which the nasopharyngeal test becomes negative (pooling samples obtained at weeks 1 and 5). Cytokine concentrations were significantly higher in breastmilk samples of mothers whose RT-PCR remained positive at postpartum week 3 (dark blue box) compared with the control group. Trends also pointed toward higher concentrations in the former compared with those who became negative sooner, within the first 2 postpartum weeks (yellow box). The values are expressed as log_10_ of the concentrations (ng/L). Statistical differences on pairwise *post hoc* comparisons between the study and control groups are indicated with an asterisk (*p < 0.05; **p ≤ 0.01; ***p ≤ 0.001, Wilcoxon rank test, Bonferroni *post hoc* test).

Evolution of cytokine concentration on breastmilk samples over time showed no differences in the study group women, with the exception of IFN-γ, IL1ra, IL4, IL9 that significantly decreased between week 1 and week 5 postpartum. In the control group women, all the tested cytokines decreased over time, with the exception of IL10, IL12, and IL13 that remained stable. Time of nasopharyngeal swab to become negative influenced the cytokines pattern in breastmilk (**Figure 2**).

#### Breastmilk Chemokine Pattern

**Table 2** displays concentration of chemokines in breastmilk samples. Eotaxin, IP-10, MIP-1α, and RANTES showed higher breastmilk concentration among the study group than in the control group at week 1 postpartum; at week 5, the concentration of chemokines, except for GRO-α, was significantly higher in breastmilk samples of the study group than in the control group.

**Table 2:** Concentration* of chemokines in milk samples of study group and control group women over time.

Week 1 postpartum				Week 5 postpartum				
	STUDY GROUP (n = 36)	CONTROL GROUP (n = 45)		STUDY GROUP (n = 37)	CONTROL GROUP (n = 45)			
Immune Factors	Median (IQR)	Median (IQR)	^a^ *ρ*	Median (IQR)	Median (IQR)	^b^ *ρ*	^c^ *ρ*	^d^ *ρ*
Eotaxin	24.2 (14.6-32.9)	14.5 (4.3-23.3)	0.020	16.9 (10.4-21)	3.1 (1.8-5.3)	<0.001	<0.001	<0.001
IL-8	1919.0 (299.7-2606.6)	584.2 (234.7-1916.7)	0.137	1921.4 (213.4-2021.5)	47.7 (20.5-167.7)	<0.001	0.001	<0.001
IP-10	18.2 (7.2-53)	10.5 (4.1-18.5)	0.025	21.9 (10.0-58.3)	2.8 (1.2-11.8)	<0.001	0.750	0.002
MCP-1	2078.4 (650.8-2738.4)	892.7 (544.1-2601.7)	0.221	2010.2 (730.6-2288.2)	123.1 (53.4-732.4)	<0.001	0.027	<0.001
MIP-1α	43.9 (10.3-47.1)	13.4 (5.3-41.9)	0.040	43.4 (26.1-44.0)	2.5 (1.0-6.7)	<0.001	0.041	<0.001
MIP-1β	212.7 (81.2-344.9)	112.9 (38.3-293.6)	0.270	191.6 (88.2-204.3)	10.5 (7.3-44.3)	<0.001	0.054	<0.001
RANTES	84.5 (48.7-115.0)	44.3 (26.2-84.1)	0.032	81.0 (61.1-142.1)	18.5 (14-39.8)	<0.001	0.427	0.011
GRO-α	7.4 (4.8-8.9)	7.9 (6.6-8.5)	0.314	7.3 (4.7-8.7)	7.3 (6.0-8.1)	0.856	<0.001	<0.001

*Concentrations are expressed as ng/L, with the exception of IP-10 and GRO-α (expressed as μg/L).

a ρ: Mann-Whitney U test was used to evaluate differences in concentration of chemokines between milk samples of STUDY GROUP and CONTROL GROUP collected in week 1 postpartum.

b ρ: Mann-Whitney U test was used to evaluate differences in concentration of chemokines between milk samples of STUDY GROUP and CONTROL GROUP collected in week 5 postpartum.

c ρ: Wilcoxon signed rank test was used to evaluate differences in the concentration of chemokines of milk the samples of STUDY GROUP between week 1 and week 5 postpartum.

d ρ: Wilcoxon signed rank test was used to evaluate differences in the concentration of chemokines of the milk samples of CONTROL GROUP between week 1 and week 5 postpartum.

**Figure 3:**
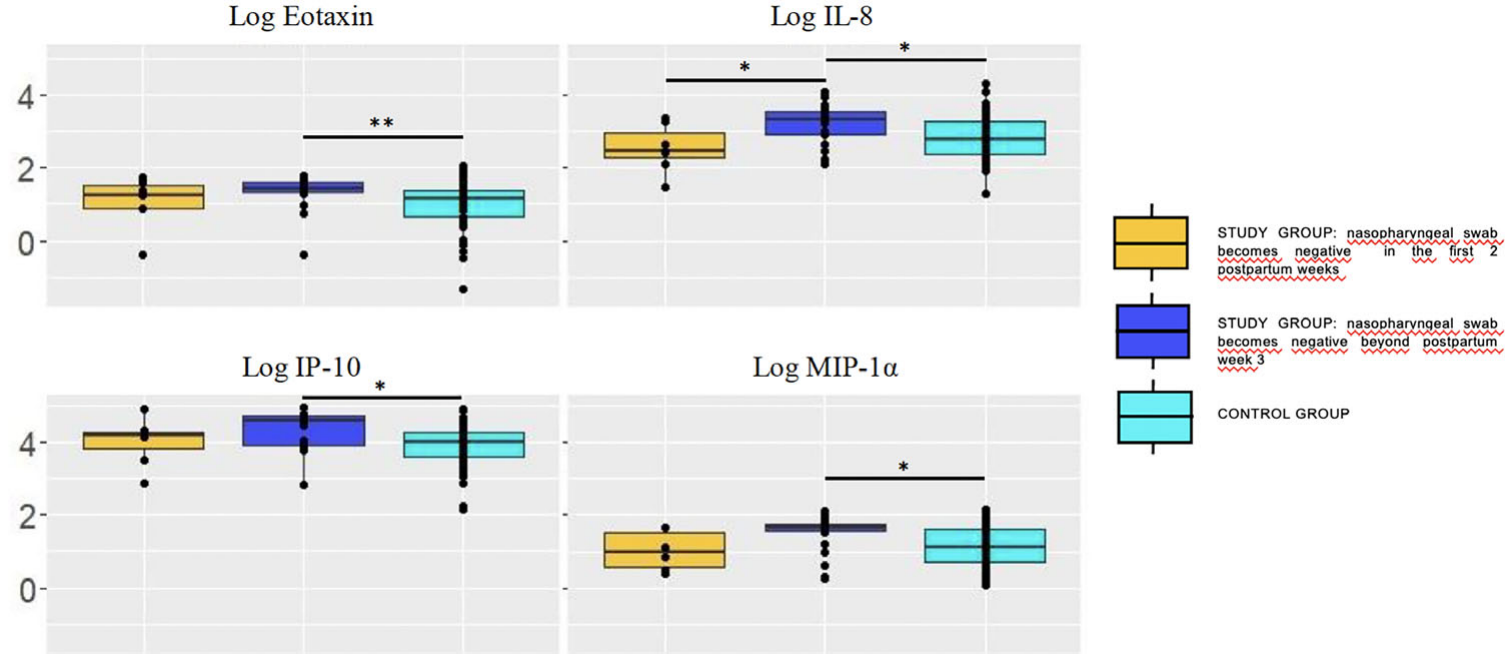
Concentration (log) of chemokines in breastmilk of study group women according to the moment in which the nasopharyngeal test becomes negative (pooling samples obtained at weeks 1 and 5). Chemokyne concentrations were significantly higher in breastmilk samples of mothers whose RT-PCR remained positive at postpartum week 3 (dark blue box) compared with the control group. Trends also pointed toward higher concentrations in the former compared with those who became negative sooner, within the first 2 postpartum weeks (yellow box). The values are expressed as log_10_ of the concentrations (ng/L), with the exception of IP-10 (μg/L). Statistical differences on pairwise *post hoc* comparisons between the study and control groups are indicated with an asterisk (*p < 0.05; **p ≤ 0.01; ***p ≤ 0.001, Wilcoxon rank test, Bonferroni *post hoc* test).

Concentration of chemokines decreased over time, it being statistically significant for most of them in the study group and for all tested chemokines in the control group. Time of nasopharyngeal swab to become negative influenced the chemokines pattern in breastmilk (**Figure 3**).

#### Breastmilk Growth Factor Pattern

**Table 3** displays concentration of growth factors in breastmilk samples. Overall, concentrations of growth factors were higher in the breastmilk samples of the study group compared with the control group women. The differences were statistically significant for basic FGF, GM-CSF, IL7, and PDGF-BB at week 1 postpartum, and for all of them, with the exception of IL-5 and EGF, in the samples collected at week 5.

**Table 3:** Concentration* of growth factors in milk samples from study group and control group women over time.

Week 1 postpartum				Week 5 postpartum				
	STUDY GROUP (n = 36)	CONTROL GROUP (n = 45)		STUDY GROUP (n = 37)	CONTROL GROUP (n = 45)			
Immune Factors	Median (IQR)	Median (IQR)	^a^ *ρ*	Median (IQR)	Median (IQR)	^b^ *ρ*	^c^ *ρ*	^d^ *ρ*
Basic FGF	64.0 (28.6-73.3)	22.5 (16.4-46.3)	<0.001	59.4 (55.1-64.3)	12.8 (9.6-21.7)	<0.001	0.394	<0.001
G-CSF	178.6 (116.1-287.1)	104.3 (68.1-224.6)	0.227	147.8 (121.9-203.5)	32.4 (12-110.9)	<0.001	0.245	<0.001
GM-CSF	13.5 (12.9-14.6)	1.4 (0.8-2.7)	<0.001	13.6 (7.8-16.1)	2 (0.8-11)	0.006	0.086	0.016
IL-5	17.4 (7.9-24.3)	23.7 (10.9-36.9)	0.321	21.1 (14.8-35.7)	20.8 (9.8-29.5)	0.540	0.275	0.612
IL-7	114.7 (33.3-147.7)	27.5 (19.3-33.4)	<0.001	113.9 (53.2-135.0)	28.2 (21.1-52.3)	<0.001	0.225	0.052
PDGF-BB	54.8 (28.7-70.7)	23.6 (14.9-31.5)	<0.001	40.9 (35.7-67.5)	23.6 (13.3-34.8)	<0.001	0.458	0.786
VEGF	11.5 (4.5-16.4)	7.6 (3.8-11.2)	0.090	10.4 (3.7-11.5)	2.7 (1.8-3.4)	<0.001	0.007	<0.001
TGF-β2	2.5 (1.7-3.0)	2.1 (1.7-2.5)	0.164	2.7 (1.7-3.0)	1.9 (1.7-2.1)	0.003	0.014	<0.001
EGF	5.6 (4.5-6.6)	5.4 (4.9-5.9)	0.768	5.4 (4.3-6.0)	5 (4.3-5.5)	0.120	<0.001	<0.001

*Concentrations are expressed as ng/L, with the exception of VEGF, TFG-β2 and EFG (expressed as μg/L).

a ρ: Mann-Whitney U test was used to evaluate differences in concentration of growth factors between milk samples of STUDY GROUP and CONTROL GROUP collected in week 1 postpartum.

b ρ: Mann-Whitney U test was used to evaluate differences in concentration of growth factors between t milk samples of STUDY GROUP and CONTROL GROUP collected in week 5 postpartum.

c ρ: Wilcoxon signed rank test was used to evaluate differences in the concentration of growth factors of the milk samples of STUDY GROUP between week 1 and week 5 postpartum.

d ρ: Wilcoxon signed rank test was used to evaluate differences in the concentration of growth factors of the milk samples of CONTROL GROUP between week 1 and week 5postpartum.

**Figure 4:**
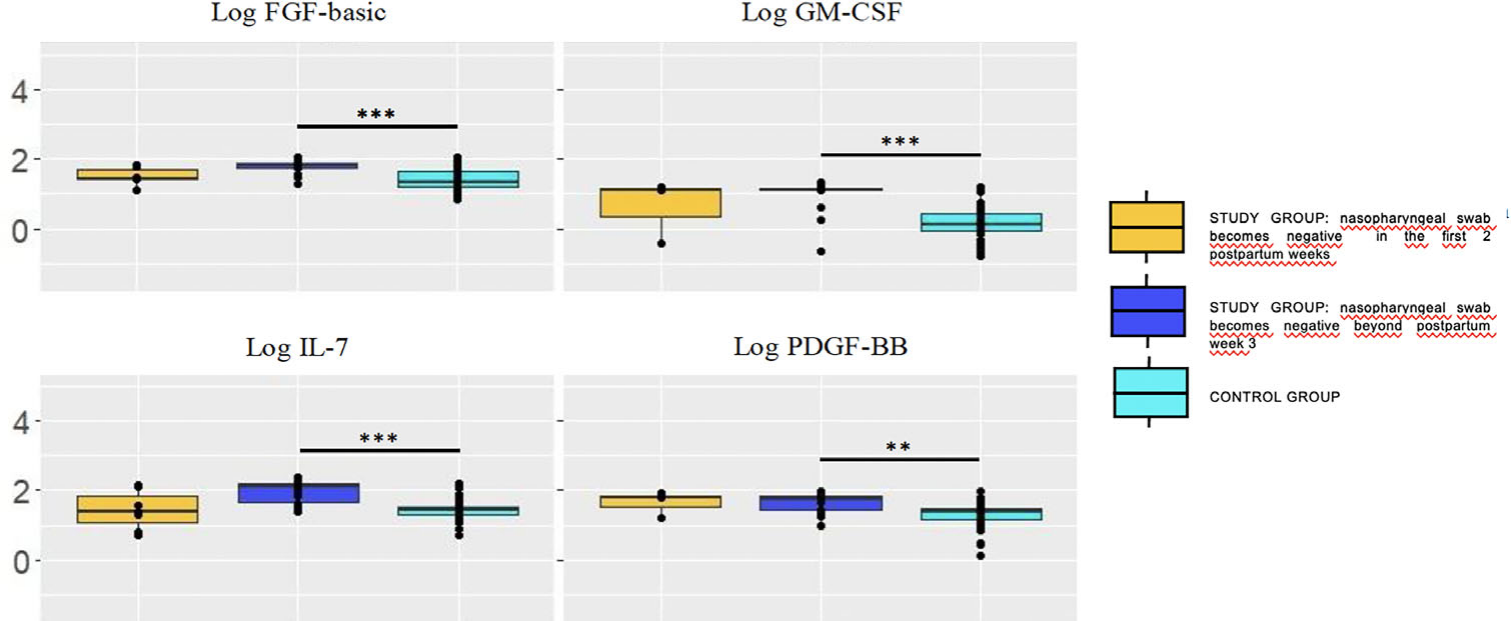
Concentration (log) of growth factors in breastmilk of study group women according to the moment in which the nasopharyngeal test becomes negative (pooling samples obtained at week 1 and 5). Growth factors concentrations were significantly higher in breastmilk samples of mothers whose RT-PCR remained positive at postpartum week 3 (dark blue box) compared with the control group. Trends also pointed toward higher concentrations in the former compared with those who became negative sooner, within the first two postpartum weeks (yellow box). The values are expressed as log_10_ of the concentrations (ng/L). Statistical differences on pairwise *post hoc* comparisons between the study and control groups are indicated with an asterisk (*p < 0.05; **p ≤ 0.01; ***p ≤ 0.001, Wilcoxon rank test, Bonferroni *post hoc* test).

Most growth factor concentrations remained stable over time in study group; in contrast, concentrations of basic FGF, G-CSF, VEGF, TGF-β2, and EGF significantly decreased from week 1 to week 5 postpartum in the control group breastmilk samples. Time of nasopharyngeal swab to become negative influenced the growth factor pattern in breastmilk (**Figure 4**).

#### Breastmilk Immunological Pattern According to Disease State

Concentration of immune factors in breastmilk samples in the SARS-CoV-2–positive women depending on the presence or not of COVID-19-related symptoms did not show relevant differences, neither in the first sampling time (lower IL-12 in symptomatic than in asymptomatic women, p<0.01) nor in the second (higher IL-15 (p<0.01) and lower GM-CSF (p<0.03) in symptomatic women).

## Discussion

This study confirms that in the asymptomatic or non-severe infected pregnant woman, breastmilk samples do not carry SARS-CoV-2 RNA. These results coincide with those found in previous studies (1–3, 7, 24–26), although a very low rate of positive milk samples (4) or isolated case reports (27–31) have also been published. This information is crucial as neonatal infection by SARS-CoV-2 is uncommon and usually asymptomatic (32–34). The lack of viral RNA in breastmilk supports its safety, and it is in accord with recent epidemiological data, as several small observational studies reported on the absence of infection in infants fed by breastmilk of SARS-CoV-2 positive woman (35, 36) or inadvertently fed with SARS-CoV-2 RNA-positive milk (29).

SARS-CoV-2 RNA seems to be widespread on surfaces from COVID-19 patient rooms (37) and, also, on the breast skin of lactating mothers (3). This suggests that milk samples may become contaminated with viral RNA when a mother and/or her neonate are positive; therefore, caution should be extreme to avoid contaminations when performing SARS-CoV-2 assessments of human milk. In addition, it must be highlighted that RT-PCR assays can only detect viral RNA but not viable infectious viruses. A detailed virological assessment of some COVID-19 cases showed that, although high concentrations of viral RNA were found both in pharyngeal and fecal samples, the virus itself could be readily isolated from throat or lung samples but not from the fecal ones (38). A study involving 64 milk samples from 18 women who had confirmed SARS-CoV-2 supported this affirmation. Only one sample had a detectable level of viral RNA but no replication-competent virus was detectable in any sample, including the sample that was positive for SARS-CoV-2 RNA (2). To date, no study has described presence of infectious SARS-CoV-2 in colostrum and milk.

The second objective of this study was the profiling of cytokines, chemokines, and growth factors in the milk samples. Overall, the concentrations of most of the immune factors analyzed were higher in the samples of positive women than in those of negative ones. This activation of innate and adaptative immune response constitutes the first line of defense against viral infections (16–18, 39).

A study involving the assessment of immunological compounds in milk produced by healthy women, found a higher co-occurrence of immune factors and higher TNFα to IL10 ratios in milk samples from healthy women with a higher level of exposure to microorganisms (40). In our study, the same was observed in the milk of SARS-CoV-2-positive women, a finding which is consistent with a wider plasticity of the immune responses.

Concentrations of most of the immune factors analyzed remained stable over time in SARS-CoV-2-positive women milk samples. In contrast, most of these compounds significantly decreased from the first to the fifth week postpartum in negative women. Previous studies have reported notably higher concentrations of several cytokines, chemokines, growth factors, and immunoglobulins in colostrum than in mature milk (13, 41–44). Although this could simply be a physiological response from the mother to infection and not enough proof for it to be protective, our findings could also suggest that, in the presence of a viral infection, the immunological profile of human milk may be adapted to provide additional infant’s protection against the maternal infection. Evidences for an immunological cross-talk between mothers and their breast-fed infants during infections, including severe viral respiratory infections, have already been provided (45, 46). In our study, positive women were either asymptomatic or suffered mild disease, therefore the evolutive profile of immunological compounds in breastmilk was related to the time when RT-PCR swabs became negative, as a marker of disease state. Mothers who later became negative for nasopharyngeal RT-PCR presented persistently higher levels of several immunological factors. This immunological profile has been described in COVID19 pathophysiology related to the severity of the infection (16–18, 39). Of note, all the newborns in the study and control group remained free of clinical signs of SARS-COV-2 infection in the first month of life.

In this study, VEGF, TGF-β2 and EGF were the most abundant growth factors found in the milk samples, independently of the SARS-CoV-2 status or the sampling time, the concentration being 50- to 100-fold higher, coinciding with those detected in healthy women as reported in a previous study (40). These three immune compounds play critical roles in infants’ health and development, including protection against infectious diseases, modulation of inflammatory processes, and establishment of food tolerance (12, 40, 47–49).

This study has several limitations. There is a lack of comparison between serum and milk immune factors profile, and the antibody levels were not assayed. The distinct profile in immune compounds in breast milk of SARS-CoV-2 positive women could not be translated into effective gastrointestinal absorption and in a functional form to impact the infant immune system. Follow-up losses in the control group could eventually modify the results. Finally, the analysis of the immune compounds was limited to two milk samples, with an interval of approximately 1 month. We took this decision after confirmation of the lack of viral RNA in any of the milk samples over time. Therefore, we considered that the observation period was adequate to see eventual evolution of the immune compounds related to mother’s infection status. On the other hand, a strength of this work is the huge variety of compounds that have been analyzed, and the systematic approach to both SARS-CoV-2 documented infection and control women.

In summary, the results of this study provide additional evidence to the safety of breastfeeding in SARS-CoV-2 infected women, as RNA was not detected in any of the milk samples tested throughout the observation period. Our results also suggest that the immune system of the infected women reacted efficiently against SARS-CoV-2 as a distinct pattern of cytokines, chemokines, and growth factors was observed in the milk samples of infected women, that persisted over time. However, this cannot be directly extrapolated to a beneficial effect in the infant. More studies are required to elucidate if this pattern only reflects the inflammatory status of the mother or if it may be linked to the development of an integration of the mother-infant immune systems, being especially suitable to protect recipient child.

## Data Availability Statement

The raw data supporting the conclusions of this article will be made available by the authors, without undue reservation.

## Ethics Statement

The studies involving human participants were reviewed and approved by Ethical committee of clinical research of La Paz University Hospital. Written informed consent to participate in this study was provided by the participants’ legal guardian/next of kin.

## Author Contributions

LS conceptualized and designed the study, participated in patient’s enrolment, data gathering and analysis, drafted the initial manuscript, and reviewed and approved the final version. AP and JR conceptualized and designed the study, and participated in data analyses, drafted the initial manuscript, and reviewed and approved the final version. FC conceptualized and designed the study, and reviewed and approved the final version of the manuscript. RG-S, ML-A, MM-P, DE-V and EC-A participated in patient’s enrolment and data gathering, and reviewed and approved the final version of the manuscript. NG-T and IC participated in sampling management and analysis, drafted the initial manuscript, and reviewed and approved the final version. CA participated in statistical and data analyses. All authors contributed to the article and approved the submitted version.

## Funding

This work was supported by Instituto de Salud San Carlos III [COV20/01046]; Ministerio de Ciencia, Innovación y Universidades (Spain) by Irma Castro predoctoral contract [BES-2017-080713] and RETICS “Maternal and Child Health and Development Network” (SAMID Network), funded by the PN I+D+i 2013-2016 (Spain), ISCIII-Sub-Directorate General for Research Assessment and Promotion and the European Regional Development Fund (ERDF) [RD16/0022].

## Conflict of Interest

The authors declare that the research was conducted in the absence of any commercial or financial relationships that could be construed as a potential conflict of interest.

## Publisher’s Note

All claims expressed in this article are solely those of the authors and do not necessarily represent those of their affiliated organizations, or those of the publisher, the editors and the reviewers. Any product that may be evaluated in this article, or claim that may be made by its manufacturer, is not guaranteed or endorsed by the publisher.

## References

[1] Marín Gabriel MA Cuadrado I Álvarez Fernández B González Carrasco E Alonso Díaz C Neo-COVID-19 Research Group. Multicenter Spanish Study Found No Incidences of Viral Transmission in Infants Born to Mothers With COVID-19. Acta Paediatr (2020) 109(11):2302–8. 10.1111/apa.15474 PMC7404522 32649784

[2] Chambers CD Krogstad P Bertrand K Contreras D Tobin NH Bode L. Evaluation of SARS-CoV-2 in Breastmilk From 18 Infected Women. JAMA (2020) 324(13):1347–8. 10.1001/jama.2020.15580 PMC7439212 32822495

[3] Pace RM Williams JE Järvinen KM Belfort MB Dw Pace C Lackey KA. COVID-19 and Human Milk: SARS-CoV-2, Antibodies, and Neutralizing Capacity. (2020). 10.1101/2020.09.16.20196071. medRxiv.

[4] Bertino E Moro GE De Renzi G Viberti G Cavallo R Collaborative Research Group on SARS-CoV-2 in Human Milk. Detection of SARS-CoV-2 in Milk From COVID-19 Positive Mothers and Follow-Up of Their Infants. Front Pediatr (2020) 27:8:597699. 10.3389/fped.2020.597699 PMC7652760 33194929

[5] Cheema R Partridge E Kair LR Kuhn-Riordon K Silva AI Bettinelli ME. Protecting Breastfeeding During the COVID-19 Pandemic. Am J Perinatol (2020). 10.1055/s-0040-1714277 PMC7872205 32693415

[6] Centeno-Tablante E Medina-Rivera M Finkelstein JL Rayco-Solon P García-Casal MN Rogers L. Transmission of SARS-CoV-2 Through Breast Milk and Breastfeeding: A Living Systematic Review. Ann N Y Acad Sci (2021) 1484(1):32–54. 10.1111/nyas.14477 32860259 PMC7970667

[7] Martins-Filho PR Santos VS Santos HP Jr. To Breastfeed or Not to Breastfeed? Lack of Evidence on the Presence of SARS-CoV-2 in Breastmilk of Pregnant Women With COVID-19. Rev Panam Salud Publica (2020) 44:e59. 10.26633/RPSP.2020.59 32454808 PMC7241574

[8] Demers-Mathieu V Do DM Mathijssen GB Sela DA Seppo A Järvinen KM. Difference in Levels of SARS-CoV-2 S1 and S2 Subunits- and Nucleocapsid Protein-Reactive SIgM/IgM, IgG and SIgA/IgA Antibodies in Human Milk. J Perinatol (2020). 10.1038/s41372-020-00805-w PMC7461757 32873904

[9] Fox A Marino J Amanat F Krammer F Hanh-Holbrook J Zolla-Pazner S. Robust and Specific Secretory IgA Against SARS-CoV-2 Detected in Human Milk. iScience (2020) 23(11):101735. 10.1016/j.isci.2020.101735 33134887 PMC7586930

[10] Garofalo RP Goldman AS. Cytokines, Chemokines, and Colony-Stimulating Factors in Human Milk: The 1997 Update. Biol Neonate (1998) 4(2):134–42. 10.1159/000014019 9691155

[11] Field CJ. The Immunological Components of Human Milk and Their Effect on Immune Development in Infants. J Nutr (2005) 135:1–4. 10.1093/jn/135.1.1 15623823

[12] Dvorak B. Milk Epidermal Growth Factor and Gut Protection. J Pediatr (2010) 156(2 Suppl):S31–5. 10.1016/j.jpeds.2009.11.018 PMC2852639 20105663

[13] Garofalo RP. Cytokines in Human Milk. J Pediatr (2010) 156:S36–40. 10.1016/j.jpeds.2009.11.019 20105664

[14] Dawod B Marshall JS. Cytokines and Soluble Receptors in Breast Milk as Enhancers of Oral Tolerance Development. Front Immunol (2019) 10:16. 10.3389/fimmu.2019.00016 30723472 PMC6349727

[15] Ruiz L Fernández L Rodríguez JM. Immune Factors in Human Milk. In Human Milk: Sampling and Measurement of Energy-Yielding Nutrients and Other Macromolecules. McGuire M O’Connor D, editors. London: Academic Press (2020).

[16] Azkur AK Akdis M Azkur D Sokolowska M van de Veen W Brüggen MC. Immune Response to SARS-CoV-2 and Mechanisms of Immunopathological Changes in COVID-19. Allergy (2020) 75(7):1564–81. 10.1111/all.14364 PMC7272948 32396996

[17] Chatterjee SK Saha S Munoz MNM. Molecular Pathogenesis, Immunopathogenesis and Novel Therapeutic Strategy Against COVID-19. Front Mol Biosci (2020) 7:196. 10.3389/fmolb.2020.00196 32850977 PMC7431665

[18] Choudhary S Sharma K Silakari O. The Interplay Between Inflammatory Pathways and COVID-19: A Critical Review on Pathogenesis and Therapeutic Options. Microb Pathog (2020) 150:104673. 10.1016/j.micpath.2020.104673 33278517 PMC7709793

[19] Farquhar C Mbori-Ngacha DA Redman MW Bosire RK Lohman BL Piantadosi AL. CC and CXC Chemokines in Breastmilk are Associated With Mother-to-Child HIV-1 Transmission. Curr HIV Res (2005) 3:361–9. 10.2174/157016205774370393 16250882

[20] Bosire R Guthrie BL Lohman-Payne B Mabuka J Majiwa M Wariua G. Longitudinal Comparison of Chemokines in Breastmilk Early Postpartum Among HIV-1-Infected and Uninfected Kenyan Women. Breastfeed Med Off J Acad Breastfeed Med (2007) 2:129–38. 10.1089/bfm.2007.0009 PMC3381953 17903098

[21] Walter J Ghosh MK Kuhn L Semrau K Sinkala M Kankasa C. High Concentrations of Interleukin 15 in Breast Milk are Associated With Protection Against Postnatal HIV Transmission. J Infect Dis (2009) 200:1498–502. 10.1086/644603 PMC2811259 19835475

[22] de Quental OB França EL Honório-França AC Morais TC Daboin BEG Bezerra IMP. Zika Virus Alters the Viscosity and Cytokines Profile in Human Colostrum. J Immunol Res (2019) 2019:9020519. 10.1155/2019/9020519 31828175 PMC6885239

[23] Rabe T Lazar K Cambronero C Goelz R Hamprecht K. Human Cytomegalovirus (HCMV) Reactivation in the Mammary Gland Induces a Proinflammatory Cytokine Shift in Breast Milk. Microorganisms (2020) 8(2):289. 10.3390/microorganisms8020289 PMC7074878 32093317

[24] Lackey KA Pace RM Williams JE Bode L Donovan SM Järvinen KM. SARS-CoV-2 and Human Milk: What Is the Evidence? Matern Child Nutr (2020) 16(4):e13032. 10.1111/mcn.13032 32472745 PMC7300480

[25] Yang N Che S Zhang J Wang X Tang Y COVID-19 Evidence and Recommendations Working Group. Breastfeeding of Infants Born to Mothers With COVID-19: A Rapid Review. Ann Transl Med (2020) 8(10):618. 10.21037/atm-20-3299 32566555 PMC7290644

[26] Chen L Li Q Zheng D Jiang H Wei Y Zou L. Clinical Characteristics of Pregnant Women With Covid-19 in Wuhan, China. N Engl J Med (2020) 382(25):e100. 10.1056/NEJMc2009226 32302077 PMC7182016

[27] Groß R Conzelmann C Müller JA Stenger S Steinhart K Kirchhoff F. Detection of SARS-CoV-2 in Human Breastmilk. Lancet (2020) 395(10239):1757–8. 10.1016/S0140-6736(20)31181-8 PMC7241971 32446324

[28] Hinojosa-Velasco A de Oca PVB García-Sosa LE Mendoza-Durán JG Pérez-Méndez MJ Dávila-González E. A Case Report of Newborn Infant With Severe COVID-19 in Mexico: Detection of SARS-CoV-2 in Human Breast Milk and Stool. Int J Infect Dis (2020) 100:21–4. 10.1016/j.ijid.2020.08.055 PMC7449937 32860950

[29] Lugli L Bedetti L Lucaccioni L Gennari W Leone C Ancora G. An Uninfected Preterm Newborn Inadvertently Fed SARS-CoV-2-Positive Breast Milk. Pediatrics (2020) 146(6):e2020004960. 10.1542/peds.2020-004960 32843439

[30] Costa S Posteraro B Marchetti S Tamburrini E Carducci B Lanzone A. Excretion of SARS-CoV-2 in Human Breast Milk. Clin Microbiol Infect (2020) 26(10):1430–2. 10.1016/j.cmi.2020.05.027 PMC7266588 32502644

[31] De Rose DU Piersigilli F Ronchetti MP Santisi A Bersani I Study Group of Neonatal Infectious Diseases of The Italian Society of Neonatology (SIN). Novel Corornavirus Disease (COVID-19) in Newborns and Infants: What We Know So Far. Ital J Pediatr (2020) 46(1):56. 10.1186/s13052-020-0820-x 32349772 PMC7190200

[32] Fernández Colomer B Sánchez-Luna M de Alba Romero C Alarcón A Baña Souto A Camba Longueira F. Neonatal Infection Due to SARS-CoV-2: An Epidemiological Study in Spain. Front Pediatr (2020) 8:580584. 33194912 10.3389/fped.2020.580584 PMC7644848

[33] White A Mukherjee P Stremming J Sherlock LG Reynolds RM Smith D. Neonates Hospitalized With Community-Acquired SARS-CoV-2 in a Colorado Neonatal Intensive Care Unit. Neonatology (2020) 117(5):641–5. 10.1159/000508962 PMC7316651 32498065

[34] Wei M Yuan J Liu Y Fu T Yu X Zhang ZJ. Novel Coronavirus Infection in Hospitalized Infants Under 1 Year of Age in China. JAMA (2020) 323(14):1313–4. 10.1001/jama.2020.2131 PMC7042807 32058570

[35] Walker KF O’Donoghue K Grace N Dorling J Comeaud JL Li W. Maternal Transmission of SARS-COV-2 to the Neonate, and Possible Routes for Such Transmission: A Systematic Review and Critical Analysis. BJOG (2020) 127(11):1324–36. 10.1111/1471-0528.16362 PMC7323034 32531146

[36] Pereira A Cruz-Melguizo S Adrien M Fuentes L Marin E Forti Z. Breastfeeding Mothers With COVID-19 Infection: A Case Series. Int Breastfeed J (2020) 15(1):69. 10.1186/s13006-020-00314-8 32770999 PMC7414278

[37] Marotz C Belda-Ferre P Ali F Das P Huang S Cantrel K. Microbial Context Predicts SARS-CoV-2 Prevalence in Patients and the Hospital Built Environment. (2020). 10.1101/2020.11.19.20234229. medRxiv.

[38] Wölfel R Corman VM Guggemos W Seilmaier M Zange S Müller MA. Virological Assessment of Hospitalized Patients With COVID-2019. Nature (2020) 581(7809):465–9. 10.1038/s41586-020-2196-x 32235945

[39] Tay MZ Poh CM Rénia L MacAry PA Ng LFP. The Trinity of COVID-19: Immunity, Inflammation and Intervention. Nat Rev Immunol (2020) 20(6):363–74. 10.1038/s41577-020-0311-8 PMC7187672 32346093

[40] Ruiz L Espinosa-Martos I García-Carral C Manzano S McGuire MK Meehan CL. What’s Normal? Immune Profiling of Human Milk From Healthy Women Living in Different Geographical and Socioeconomic Settings. Front Immunol (2017) 8:696. 10.3389/fimmu.2017.00696 28713365 PMC5492702

[41] Chollet-Hinton LS Stuebe AM Casbas-Hernandez P Chetwynd E Troester MA. Temporal Trends in the Inflammatory Cytokine Profile of Human Breastmilk. Breastfeed Med (2014) 9(10):530–7. 10.1089/bfm.2014.0043 PMC4267123 25380323

[42] Moles L Manzano S Fernández L Montilla A Corzo N Ares S. Bacteriological, Biochemical, and Immunological Properties of Colostrum and Mature Milk From Mothers of Extremely Preterm Infants. J Pediatr Gastroenterol Nutr (2015) 60(1):120–6. 10.1097/MPG.0000000000000560 25207476

[43] Collado MC Santaella M Mira-Pascual L Martínez-Arias E Khodayar-Pardo P Ros G. Longitudinal Study of Cytokine Expression, Lipid Profile and Neuronal Growth Factors in Human Breast Milk From Term and Preterm Deliveries. Nutrients (2015) 7(10):8577–91. 10.3390/nu7105415 PMC4632435 26492267

[44] Trend S Strunk T Lloyd ML Heen Kok C Metcalfe J Geddes DT. Levels of Innate Immune Factors in Preterm and Term Mother’s Breast Milk During the 1st Month Postpartum. Br J Nutr (2016) 115(7):1178–93. 10.1017/S0007114516000234 26891901

[45] Bryan DL Hart PH Forsyth KD Gibson RA. Immunomodulatory Constituents of Human Milk Change in Response to Infant Bronchiolitis. Pediatr Allergy Immunol (2007) 18(6):495–502. 10.1111/j.1399-3038.2007.00565.x 17680907

[46] Riskin A Almog M Peri R Halasz K Srugo I Kessel A. Changes in Immunomodulatory Constituents of Human Milk in Response to Active Infection in the Nursing Infant. Pediatr Res (2012) 71(2):220–5. 10.1038/pr.2011.34 22258136

[47] Veldhoen M Hocking RJ Atkins CJ Locksley RM Stockinger B. TGFbeta in the Context of an Inflammatory Cytokine Milieu Supports *De Novo* Differentiation of IL-17-Producing T Cells. Immunity (2006) 24:179–89. 10.1016/j.immuni.2006.01.001 16473830

[48] Verhasselt V. Neonatal Tolerance Under Breastfeeding Influence: The Presence of Allergen and Transforming Growth Factor-β in Breast Milk Protects the Progeny From Allergic Asthma. J Pediatr (2010) 156:S16–20. 10.1016/j.jpeds.2009.11.015 20105659

[49] Penttila IA. Milk-Derived Transforming Growth Factor-β and the Infant Immune Response. J Pediatr (2010) 156:S21–5. 10.1016/j.jpeds.2009.11.016 20105660

